# Regulatory framework in Pakistan: situation analysis of medicine quality and future recommendations

**DOI:** 10.1186/s40545-019-0184-z

**Published:** 2019-09-11

**Authors:** Huma Rasheed, Ludwig Hoellein, Khalid Saeed Bukhari, Ulrike Holzgrabe

**Affiliations:** 1 Institute of Pharmacy and Food Chemistry, University of Wuerzburg, am Hubland, 97074 Wuerzburg, Germany; 2 Institute of Pharmaceutical Sciences, University of Veterinary and Animal Sciences, Lahore, Pakistan; 3 USP Promoting the Quality of Medicines (PQM) Program, U.S. Pharmacopeial Convention, 12601 Twinbrook Parkway, Rockville, MD 20852-1790 USA

**Keywords:** Pakistan, Drug regulatory authority, Pharmaceutical regulation, Substandard, Falsified, Poor-quality

## Abstract

**Background:**

Quality issues in pharmaceuticals are identified as a huge global and public health problem, especially with reference to low- and middle-income countries like Pakistan. The 2011 “Fake Drug Crisis” acted as a driving force to reform the regulatory structures of the country and for establishing the autonomous “Drug Regulatory Authority of Pakistan”. Despite the fact that Pakistan possesses a huge pharmaceutical industry, there is a severe dearth of published literature and scientific evidence for the country regarding medicine quality and the prevalence of counterfeit and low-quality products, respectively.

**Aims and objectives:**

This narrative review covers relevant features of the regulatory framework for pharmaceuticals in Pakistan, its national pharmaceutical industry, as well as a compilation and analysis of published literature for documentation of the country’s situation regarding the overall quality of medicines.

**Methods:**

Available data including scientific publications on the quality of pharmaceuticals in peer reviewed journals, research reports, notifications, and alerts issued by the World Health Organization and other agencies were accessed and compiled. Post graduate dissertations were used to represent unpublished research data and drug safety alerts issued from the local Pakistan authority were analysed to assess the type and number of quality failures reported for pharmaceuticals.

**Results:**

It could be clearly shown that there is negligible scientific data available on the issue of medicine quality in Pakistan. The anticipated number of 40–50% of poor-quality drugs in Pakistan cannot be defended by data available from the literature. Accessible technologies and strategies used in recent years at global level, especially in developing countries, were also reviewed and recommendations are devised for Pakistan to combat the fight against poor-quality medicines.

**Conclusion:**

The case reports, investigations, and general data listed for Pakistan suggest the need of strengthening regulatory systems for premises and GMP inspections, analytical laboratories, as well as an overall capacity building in the field of unravelling and controlling substandard and falsified medicines. It is proposed that well-planned and properly funded studies need to be carried out for collecting critical statistics regarding the prevalence of substandard and falsified medicines in Pakistan.

## Background

Quality issues in pharmaceuticals have been identified as a huge global public health problem since many years [1–5], especially with reference to low- and middle-income countries (LMICs) like Pakistan [3, 6, 7]. Despite the fact that the country possesses a huge pharmaceutical industry, there is a severe dearth of published literature and scientific evidence on the quality of medicines.

The main objective of this review is to compile and analyse scientific publications, reports, and other published evidence which can be helpful in documenting the situation of the country regarding medicine quality and the prevalence of substandard and/or falsified medicinal products. This article also covers important features of the regulatory framework in Pakistan and its pharmaceutical industry, respectively. Recommendations are devised for the country to combat the fight against poor-quality medicines. These recommendations are based on the findings of the review and incorporate information on globally tested cost-effective technologies especially with regard to LMICs.

### The situation in Pakistan

The following section aims to describe the complexity of the current situation in Pakistan regarding both the pharmaceutical and the health sector. Pakistan has a large pharmaceutical manufacturing sector and a huge population with poor health indicators in many respects. In 2012, an autonomous Drug Regulatory Authority was formed because the previous system could not provide any effective pharmaceutical regulation. This led to a complex and highly demanding situation. This section describes the shift towards a new, more stringent, and better equipped system and also identifies the drivers and barriers of change within the whole process. It includes published data and reports on the quality of medicines circulating in Pakistan in order to separate myths from facts.

### Overview

Pakistan is among the nations that recently adopted the concept of an autonomous Drug Regulatory Authority [8, 9] and the country is currently developing and implementing a comprehensive national pharmacovigilance system [8–11]. Pakistan has undergone many reforms and policy changes in the past few years for ensuring the delivery of safe and efficacious medicines to the people. In November 2018, the country has acquired a full membership status to the World Health Organization’s (WHO) Programme for International Drug Monitoring (WHO-PIDM). This concept was established in 1968; the main purposes include developing a pharmacovigilance system in member countries and coordination at national and international level for timely intimating on any medicine safety alerts. With full membership status, Pakistan will have access to the respective WHO databases “VigiBase” and “VigiLyze” for performing signal detection and signal strengthening [12] and for being able to access global data for evaluating national reports.

Resource limitation is a key reason that results in poor regulatory controls particularly with respect to analytical testing of medicines. To ensure a constant delivery of quality medicines to its population, Pakistani regulators and researchers need to be informed on the affordable technologies and strategies related to implementing cost-effective policies. This synthesis can act as a stepping stone for reforming Pakistan’s regulatory systems and facilitate the delivery of safe and efficacious medicines to the population.

### The country and its population

Pakistan is a lower-middle income country [13] situated in the west of the Indian Subcontinent, having the sixth largest population of the world with over 207 million inhabitants [14]. 43.4% are below 15 years, and 3.5% are above 65 years of age [15]. In the years 2015 and 2016, the per capita public expenditure on health was 45 US-Dollar (US-$), whereby the WHO benchmark is 86 US-$ [16, 17]. Within the last 10 years [16], Pakistan has not achieved the WHO benchmark of spending 6% of the Gross Domestic Product on health. According to National Health Accounts (2015–16), 63% of total health expenditure is contributed by out-of-pocket expense, whereas the provincial health departments and district government cover only 16 and 4.6% of total health expenditure, respectively [17]. The infant, neonatal, and under-5 mortality rates per 1000 live births for 2015 were recorded as 64.6, 46.3, and 79.5, respectively, whereas maternal mortality was 178 per 100,000 [18]. The number of deaths due to infectious diseases and respiratory infections constitutes a major portion of the total mortality. The current health system and its regulation have long been criticized for the lack of infrastructure, incompetence, and organizational weaknesses [11, 19–21]. The absence of pharmacists from the health care system and the lack of sufficient regulatory controls have resulted in the mishandling, misuse, and overuse of pharmaceuticals including antibiotics in the country [21].

### Pharmaceutical sector of Pakistan

The country holds annual pharmaceutical sales of 3.1 billion US-$ with systematic anti-infectives followed by drugs used for gastrointestinal and metabolic disorders representing the major categories of sold finished pharmaceuticals [22]. The larger share (about 60%) of sales goes to domestically produced medicines with 95% of the Active Pharmaceutical Ingredients (APIs) being imported from abroad [22].

According to the press release issued by the “Drug Regulatory Authority of Pakistan” (DRAP) on 26th January 2019, Pakistan has 647 actively operating drug manufacturing licenses, and 6440 medicines were registered in the year of 2018 [23]. The manufacturing licenses cover formulation, basic and semi-basic manufacturing (raw material manufacturing), as well as repacking. However, the list of importers of finished pharmaceutical products including mainly biologicals, vaccines, anticancers, newly approved medicines, contrast media, etc., exceed the number of pharmaceutical manufacturers. Out of all licensed manufacturing units in Pakistan, none has been approved by the United States Food and Drug Administration (FDA) which is in strong contrast to the neighbours India and Bangladesh [24]. Only one pharmaceutical company actually holds a Good Manufacturing Practice (GMP) certification issued by the European Medicines Agency [25] and accreditation issued by the Medicines and Healthcare Products Regulatory Agency of the UK (MHRA) for solid dosage form manufacturing [26].

In early 2018, moxifloxacin tablets produced by Getz Pharma Pvt., Ltd. achieved the status of a “first-ever WHO prequalified pharmaceutical product from Pakistan” [27]. Nationally manufactured pharmaceuticals are exported to other countries, mainly to Jordan (80%), Africa, and the Middle East [28]. A Central Research Fund (CRF) is operated under the “Drug (licensing, registration, and advertising) rules” from 1976 [29], according to which 1% of the gross profit of pharmaceutical companies before tax deduction is deposited to the government for supporting research of public and national interests [22, 30]. According to the figures reported in 2007, CRF amounted to 467 million Pakistani rupees (3.3 million US-$), with the estimates of 75–85 million Pakistani rupees (0.5–0.6 million US-$) collected per annum [31]. However, since the beginning of an actual approval of projects under this fund in 2001, only a meagre amount has been consumed which caters to a handful of projects showing underutilization of funds, lack of planning, and poor execution of policies [31].

### Identification of medicine quality as a prevailing health sector crisis

Poor-quality pharmaceuticals and medicine supply shortages were identified as major challenges in distributing and accessing essential medicines during the humanitarian crisis following the 2005 earthquake [32, 33], floods (2010), and internally displaced people (2011). In Pakistan, the issue has gained national as well as international attention after two major incidences of poor-quality medicines claimed lives of hundreds of people in 2011 and 2012 [34–36]. The first incidence is the case of contaminated cardiovascular drugs in December 2011 which claimed more than 230 lives (“The Fake Drug Crisis”) [34]. This incidence led to the establishment of the autonomous Drug Regulatory Authority of Pakistan (DRAP) [8, 37] and also became a driving force for improving the regulatory structures of the country both at provincial and federal levels. These changes are detailed in the latter sections of this article. In November 2012, another major case of medicine quality failure occurred, causing death of hundreds of people after ingesting contaminated cough syrup [36]. It is important to note that the public sector medicine quality control laboratories were unable to identify the presence of an erroneous substance (pyrimethamine) in the cardiovascular drug in the first case, and similarly high amounts of toxic levomethorphan were not identified timely in substandard dextromethorphan cough syrup samples [34, 38]. Through these incidences, the inadequacy of the system became evident and quality issues of pharmaceuticals were gradually identified as a major health system concern in Pakistan.

### Regulatory infrastructure for pharmaceuticals

In Pakistan, medicine licensing, manufacturing, registration, pricing, imports, and exports are dealt by the federal government, whereas distribution and sales are regulated by the respective provincial governments [10]. International experts consider this decentralized regulatory control as a structural weakness of the system [38]; the situation worsened on devolution of drug regulatory powers from the federal government to the provinces under the 18th amendment of the constitution of Pakistan [20]. The decision was later revoked by issuance of a “no objection” certificate by provinces through the Council of Common Interest [37]. Along with drug regulation, the Higher Education Commission is another example of the organizational body that remained under the federal body [37].

DRAP was formed in November 2012 with enforcement of the DRAP act [8]. DRAP functions as an autonomous body under the Ministry of National Health Services [16]. The new organizational structure of DRAP consists of eight technical and five supportive divisions. The department of quality assurance has five field offices supported by federal drug inspectors, assistant drug controllers, and an appellate board. The other seven technical divisions include registration, medical devices, biological drugs, controlled drugs, pharmacy services, health & over-the-counter, costing, and pricing [39]. The pharmacy services division covers pharmacovigilance, clinical trials, regulation of contract research organizations, and research. The regulatory functions enforced through DRAP in the short period of 7 years (2012–2019), in comparison to the working of previous regulatory entity Drug Control Organization (DCO), are shown in Table 1 which also depicts the basic differences between the two organizations.

**Table 1:** Comparison of pre and post DRAP scenario of pharmaceutical regulations in Pakistan

Parameter	Pre DRAP status	Current status under DRAP
Name	Drug Control Organization	Drug Regulatory Authority
Established	1976	2012
Administrative status	Under MoH	Autonomous (financial, technically and administrative)
Human resource (Total No. of technical staff)	36	185 technical personnel, including predominantly pharmacists, pharmacologists, physicians, chemists, and microbiologists
Financial system	Dependent on MoH for budgetary allocation and funding	Self-sustained system with minimal dependence on the external funding through federal government resulting in a stronger independent position and autonomous structure
Organizational structure	• Two divisions including premises licensing, Quality Assurance, and drug registration (market authorization) • Two drug controllers reporting to Director	• 13 divisions, five thereof related to non-technical areas • Financial and administrative autonomy with secretary health as chair to policy board and Chief Executive Officer of DRAP as secretary policy board
Infrastructure	• Four provincial offices run by deputy director general, (DDGs), Federal Drugs inspectors (FIDs), and Assistant Drug Controllers (ADC) • Limited staff and authorities with most of the decisions referred back to central offices in Islamabad • Other infrastructure includes two central Drug testing Laboratories (DTLs) and a National Biological Control Laboratory • A Federal Drug Surveillance Laboratory (FSDSL) was shifted to health ministry outside the sphere DCO earlier to the establishment of DRAP	• The current provincial offices are run under additional directors with the team of twenty-five FIDs, Assistant Director Quality Assurance, and Deputy Director Quality Assurance • A higher number of technical staff with more autonomy and powers results in swift decision making • Provincial level issuance of import/export permissions, API/raw material import, sampling, inspection • assistance in customs release • With respect to working CDL has shifted to full compendial testing with the exception of a few tests including impurity testing • FDSL is reverted to its status as component of DRAP and now steps are being taken for its functional role.
Market authorization procedures	• Drug registration applications were placed in a meeting of the product registration board without any structured evaluation procedure (e.g. at times, the agenda distribution used to take place merely an hour before the meeting)	• All applications are submitted as Common technical Document (CTD) as per internationally accepted format which are subjected to a comprehensive evaluation and review procedure [40, 41] implemented through a discrete evaluation cell • Facilitation of USP-PQM in capacity building
Capacity building opportunities and their outcome and liaison with international agencies	• No formal liaison with international agencies was established.	• Hands-on support by the United States Pharmacopoeia (USP) and USAID since inception of DRAP • The liaison was a driver to implementation of CTDs [40, 41], GMP standards, Quality Management Systems (QMS), membership to Pharmaceutical Inspection Corporation Scheme (PICS), pharmacovigilance and achieving of global bench marking (GBM) • Two phases of QMS have already been completed by the end of 2017 [42]
PV status with respect to International Drug Monitoring	No status	Observer status to full Member
WHO’s National Regulatory System Global benchmarking Tool (GBT) grading on Maturity Level (ML) 1–4 [43]	No assessment	• Maturity Level status with “Reactive approach” was documented in the initial assessment done in 2014 which was shifted to “Proactive approach” in the second assessment (2017) • A third assessment is planned in 2019 for attainment of Maturity Level 3 (Stable Formal System approach) compliance certification [44] • NRAs with ML3 and ML4 compliance status are regarded as WHO listed authorities (WLAs) [43]
CRF liquidation and research initiatives	Only a meagre amount has been used and no concrete research priorities, policies or plans were present	The area is still unaddressed

DRAP started with clear emphasis on recruiting and developing highly skilled regulatory personnel, modernizing the systems, establishing mechanisms for pharmacovigilance, and upgrading equipment, human resources, and operational systems of the DTLs [10, 16, 28]. However, budgetary allocations are the main hindrances faced by DRAP [28]. Regulatory reforms are enacted by reinforcing infrastructure and human resource development as well as establishing external linkages and accreditations to increase the credibility and efficiency of the newly formed DRAP [10, 28, 45]. A list of 60,000 registered medicines has been made accessible on the DRAP website and the organization aims for adopting a 2D bar coding system as a measure to combat falsified medicines [46]. Recently, DRAP has initiated the task of digitization of its data for more transparency and clarity in supply chain management procedures and regulatory controls [47].

A short comparison of DRAP with one of the stringent regulatory systems helps to understand what more can be done and to identify the areas that are not addressed or lack an appropriate strategy. Here, the German Regulatory Agency, i.e. the Federal Institute for Drugs and Medical Devices (Bundesinstitut für Arzneimittel und Medizinprodukte, BfArM) is taken for comparison. It can be assessed from the organizational scheme of BfArM that research and product specialization are important components of its structural design: Separate departments work on identifying and tracking falsified medicines, medicine shortages, as well as parallel imports.

Moreover, a dedicated research division has been established that includes pharmacogenomics, pharmacoepidemiology, biostatistics and specialized pharmacology, and experimental neuropsychopharmacology. No such research division is present in DRAP. Another important difference between the two organizational strategies is the product specialization. Within the BfArM, medicines are allotted to the respective regulatory sections in accordance with their clinical category, so that e.g. a special commission works for the market authorization of paediatric medicines, whereas in DRAP, only the specialized therapeutic and pharmaceutical groups like anticancers and biologicals are dealt by specialized sections.

Though the human resource investment in DRAP is far more than DCO, still an involvement of more adequately trained technical force in appropriate number is needed. For comparison, BfArM works under a complex system split into 12 main divisions with 49 sections staffed by around 1000 employees. The structure of other stringent regulatory structures like FDA or Health Canada can also be reviewed to plan the future growth of DRAP.

Provision of Drug Testing Laboratories (DTLs) with adequate quality control systems and capacities is crucial for ensuring the surveillance and control of pharmaceuticals. The current testing capacity available as public sector (both federal and provincial) laboratories in Pakistan includes twelve DTLs (two central laboratories working in Karachi and Islamabad) [9], an appellate laboratory in Islamabad, five DTLs in the province of Punjab, and one in each of the remaining provinces. One DTL is located in Azad Jammu and Kashmir. A Federal Drug Surveillance Laboratory (Islamabad) is also in its developmental phase.

Three public sector DTLs are certified by the 17,025 standard of the International Organization for Standardization (ISO), whereas none is prequalified by the WHO. The Central DTL in Karachi has undergone preliminary assessments for accreditation from the WHO. One private laboratory in Pakistan gained WHO prequalification in 2014, followed by a voluntary withdrawal after 2 years [48]. Among the other drug testing facilities in the country are the Punjab Drug Testing Laboratory and Research Centre (PDTRC) and the Punjab Forensic Sciences Agency. PDTRC has recently acquired the status of Pakistan’s first WHO Accredited Medicines Quality Control Laboratory and already had ISO17025 certification.

### Interventions for delivery of quality medicines

Pakistan has been fighting the menace of poor-quality medicines since a long time. In 1975, the generic policy enacted through the Drugs (Generic Names) Act (XXIV of 1972) was repealed as a consequence of suspending 38 pharmaceutical companies for producing substandard medicines [28]. Fines (100,000-10,00,000 Pakistan Rupees, equivalent to 707–7067 US-$) and legal punishments (5–10 years of imprisonment) were enforced in the Drugs Act in 1976 as a deterrent to falsification as well as sale and manufacturing of medicines without licensing and market authorization [49]. In 2005, along with post-earthquake rescue operations, the WHO established a drug distribution network with early detection and rectification of quality and supply failures in the affected areas [33]. The WHO prequalification program was adopted [50] and accepted countrywide by a number of organizations improving the delivery of quality medicines. In a local study, no significant benefit was found in terms of time taken for smear conversion for the 15–20% more expensive internationally quality assured medicines when compared with locally produced multiple drug resistant tuberculosis medicines purchased through the medicine prequalification program [51]. In this way, the new control strategies have evolved, involving the interventions at system level rather than being limited to punishments and penalties. However, regarding substandard medicines, DRAP has reported the suspension of 89 market authorizations and cancelled the licenses of 18 companies during the 4 years period of 2013–17 [52].

Other notable interventions include the establishment of a Provincial Quality Control Unit (PDCU) of Punjab. In 2017, PDCU has initiated the dissemination of information on the failed samples of medicines to public and health professionals through its web portal [53] and a monthly newsletter [54]. PDCU has also instituted Clinical Pharmacist and Pharmacovigilance officers in health care facilities up until the district level. These are specially trained pharmacist that are involved in the implementation of an online clinical and administrative reporting system called Medicines Surveillance System. One of the prime objectives of this system is to ensure timely reporting of adverse drug reactions and misadventures as well as adverse outcomes related to medicine use in the public sector supply chain. The provincial initiative is important in the context that the early identification of any incidence like the case of contamination of cardiovascular drug can be timely identified and controlled in the massive public sector medicines supply system. As discussed in the recommendations section, other countries like Rwanda have also produced a large number of trained personnel to carry out pharmacovigilance activities to ensure early detection of substandard and falsified (SF) medicines in the supply chain [55].

### The prevalence of SF medicines in Pakistan

The most quoted figure for the prevalence of poor-quality medicines in Pakistan is 40–50% [56]. However, this figure has been criticized for lacking objective data in another publication on overall drug quality from Pakistan [57]. Limited information is available regarding the failure rates documented by public sector DTLs of Pakistan.

Out of 9089 samples from the public sector hospitals which were received within three months in 2017, the DTL in Lahore (the provincial DTL) has reported 3.3% (301 out of 9089) as out-of-specification (substandard) products [58]. A report from DRAP published in a local newspaper reported 1% substandard and 0.2% spurious (falsified) medicines from 171,375 samples tested in the central DTL, Karachi and the provincial DTLs within the period of 33 months. A 2010 report states that 2% of approximately 60,000 samples tested in a period of two years at the public sector DTLs failed to comply with quality specifications [59]. However, the official press release of DRAP for progress during 2018 states that a total of 41,435 medicine samples was tested by the public sector DTLs in 2018 of which 92.6% were declared of standard quality, 0.1% were declared spurious (falsified) and 1.2% had no registered status [23]. No explanation was provided on the remaining 6.1% and the samples declared as substandard.

## Methods

### Published data

The case reports, studies, and publications related to SF medical products [60] (previously termed as Substandard/spurious/falsified/counterfeit medicines [61] by the WHO) and related issues reported under the term poor-quality of medicine until December 2017 were accessed through PubMed and Google search engines as well as handpicked to access a maximum of possible information. “Poor quality medicine” is referred to (i) failure to meet approved specifications like over or under dose, absence of API or failure to comply to compendial tests as well as (ii) products that are fake or imitate or are prepared without legal approval or license. All publications referred to the situation in Pakistan. The major sources of information in this regard were scientific publications in peer reviewed journals, research reports, notifications, and alerts issued by the WHO and other agencies. In the investigative analysis, the reports and publications aiming at the analysis of the reported cases of poor-quality medicines were included. These involved judicial, technical, or analytical investigations by experts, researchers, or authorities of the case of suspected poor-quality medicines.

### Media reports

As media and journalism are the main first hand sources in reporting quality issues of medicines, three such reports about Pakistan were identified in Google search and were included. Only reports from reputable agencies were included, two international media from Bloomberg and CNN were identified in the search. These reports are known because of the subsequent government’s response and the media coverage by Pakistani electronic media. The third document is from the United States Pharmacopoeia (USP) “Promoting the Quality of Medicines” program and is already a compilation of media reports over 8 years and was accessed during literature search on Google.

### Samples of unpublished data

For accessing unpublished data, few hand-picked samples of PhD and M.Phil. dissertations (from the authors own institution and/or the Higher Education Portal in Pakistan) were analyzed separately.

### Review of drug safety alerts by PDCU

The Drug Safety Alerts issued by the PDCU in their monthly newsletters issued from June–November 2018 representing data from August 2017–September 2018 were compiled and categorized for the type of reports of quality failure of medicines declared by the provincial Drug testing laboratories of Punjab Province. This information is also shared publicly through an official Facebook page administrated by PDCU here only the reports compiled in official newsletter were used.

## Results

### Published information on poor-quality medicines

The published information collected from the above defined sources can be categorized into five classes (cf. Table 2).

**Table 2:** Summary of published data on the situation regarding poor-quality of medicines in Pakistan

A. International media reports	Reference
1. Media Reports on Medicine Quality: Focusing on USAID-assisted countries (2003–2011) [16 counterfeit cases]	[63]
2. Stopping fake drugs from Pakistan is too late for victims (2012) [counterfeit drug trafficking cases]	[64]
3. Inside deadly Pakistan counterfeit drug trade (2015) [capacity of regulation and provision of quality medicines]	[65]
B. Case reports and drug alerts	
4. Contaminated drugs are held responsible for 120 deaths in Pakistan (2012) [high dose of pyrimethamine found in cardiovascular drugs isosorbide dinitrate (Isotab), claiming life of more than 120 people]	[34]
5. WHO drug alert 125 [contamination of batch J093 of Isotab (isosorbide mononitrate) for precaution against the wider circulation of the batch]	[66]
6. WHO drug alert 126 - Levomethorphan contamination in dextromethorphan cough syrup (2012) [Levomethorphan was found in API supplied by the Kanduskar Laboratories, India]	[35]
C. Analysis of cases of poor-quality medicines	
7. Epidemic of *Plasmodium falciparum* malaria involving substandard antimalarial drugs, Pakistan (2003) [generic antimalarial tablets failed the dissolution test and had high content of active ingredient].	[67]
8. Pakistan’s deadly cocktail of substandard drugs (March 2012) [chaotic transition of powers and the cases of contaminated drug]	[20]
9. Batch J093: Pathology of negligence (2013) [Judicial report of contaminated cardiovascular drug case with evaluation of the regulatory capacity and recommendations to prevent and handle such incidences in future]	[19]
D. Case referenced in scientific reviews on quality of medicine	
10. Drug regulators study global treaty to tackle counterfeit drugs (2004) [40–50%]	[56]
11. How to achieve international action on falsified and substandard medicines (2012) [Discusses the 2012 fake drug crisis as a possible medicine falsification case if proven that the faulty batch found was found out of specification in the in-house quality control testing and was deliberately allowed to be distributed to hospital]	[4]
12. Substandard drugs: a potential crisis for public health (2014)	[2]
13. The essential medicines on universal health coverage (2017) [includes fake drug crisis as the major cases of poor-quality medicines in the recent years]	[38]
E. Prevalence studies on medicine quality involving pharmaceutical analysis	
14. Pharmaceutical quality of ceftriaxone generic drug products compared with Rocephin®. [34 generics including 6 products from Pakistan were evaluated on basis of Roche standards and compendial specifications. Overall, sterility test failed for 4 samples and unknown impurity monitored by Roche was found in 5 samples in concentration range of 0.39–1.26%. 30 samples failed the clarity test by USP and 33 products had higher concentration of thiotriazinone (0.22–0.94%, limit ≤0.2%).Tricef® from Ali Gohar (Pakistan) failed the assay and content uniformity test, also showing percentage content of thiotriazinone (0.94%) and unknown impurities (1.26%) [2/6]]	[68]
15. Ofloxacin; Laboratory evaluation of the antibacterial activity of 34 brands representing 31 manufacturers available in Pakistan (2004) [3/34 did not show required antimicrobial activity]	[69]
16. Quality of ceftriaxone injections reality and resonance (2008)	[57]
17. [15.6% failure rate for ceftriaxone injection]	

### Unpublished research reports

1. Ali [62] reported the following cases of substandard pharmaceuticals sampled from various sources in Pakistan in his PhD thesis:
  - Out of 27 samples of simvastatin API from 24 different sources including China, Italy, Korea, Jordan, and Germany, two samples of Indian origin failed the assay.
  - Three API samples of cefotaxime sourced from India, Jordan, and Italy were out of specification with respect to impurity content.
  - 16% of the API samples showed high level of impurities including cefotaxime, glibenclamide, and enalapril.
  - In total, 22% of all tested oral dosage forms were out of specification.
  - 22.7% of the products failed dissolution testing including mefenamic acid and diltiazem tablets.
2. Tariq [70] conducted an analysis of ceftriaxone injections from three price categories (low, medium, and high price) sampled from hospitals and retail pharmacies in Lahore. On analyzing the samples using the respective United States Pharmacopoeia monograph and assessing the antimicrobial activity all the results were found to be within the specified limits and showed antimicrobial sensitivity against the tested pathogens.
3. An M. Phil. thesis by Khan [71] reported the assay testing of cefixime capsules from Lahore utilizing Thin Layer Chromatography (TLC) and High-Performance Liquid Chromatography (HPLC) as described within the British Pharmacopoeia. Out of 14 samples of cefixime capsules, one was identified for low content both by TLC and HPLC assay methods.

### Drug safety alerts by PDCU

**Fig. 1:**
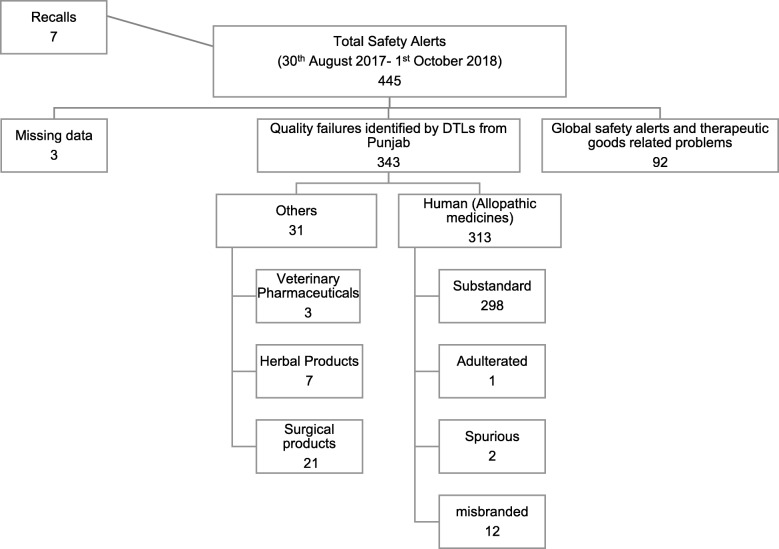
Drug safety alerts issued by Punjab Quality Control Unit in 2017 and 2018 [54]. *The terms substandard, misbranded, spurious, and adulterated medicines are according to the definitions given by the Drug Act, 1976 of Pakistan [38]

PDCU issued 445 Drug Safety Alerts (DSA) from 30 August 2017 until 1 October 2018 including 343 quality failure reports, out of which 313 reports were for medicines for human use declared as substandard, misbranded, adulterated, or spurious. The rest of the quality failures included 21 substandard and misbranded surgical products, three veterinary pharmaceuticals, and seven herbal medicines. The term “adulterated medicines” also refers to medicines found to be contaminated with foreign matter, e.g. dirt [49]. The complete data on DSAs issued by the PDCU during the studied period is shown in Fig. 1.

From the PDCU safety alert data, the major quality issues were related to substandard medicines designated for human use (Fig. 1). Apart from that, six out of seven samples of herbal medicines contained undeclared sildenafil citrate. One safety alert for Sancos Syrup (pholcodine, chlorpheniramine, and pseudoephedrine; Pfizer) was circulated for complete withdrawal of the finished product after instructions from DRAP [53]. The product was found to have issues with stability data causing a reduced shelf life [72]. Of note, the quality failure reports issued by PDCU [54] included a large number of anti-infective agents, predominantly essential beta-lactam antibiotics. Important and alarming examples include co-amoxiclav tablets (low content of clavulanic acid and failure of dissolution testing), amoxicillin suspension, ceftriaxone injection, cefixime capsules, imipenem, and meropenem injection (low API content). Most of the products analyzed by DTLs were sampled from a public-sector tender supply. Data of three DSAs was not accessible through the online version of newsletters.

Global safety alerts for communication on recently reported adverse drug reactions, therapeutic goods related problems communicated by the pharmaceutical manufacturers, and information for cancellation of licenses by DRAP constituted 92 DSAs. The seven product recalls included a recent case for valsartan containing products issued after a global alert for withdrawal of products with the carcinogenic impurity nitroso-dimethylamine [73].

## Discussion

### Published and unpublished data of medicine quality in Pakistan

This brief review of literature clearly shows that there is a negligible amount of scientific data assessing, analyzing, and discussing the topic of medicine quality in Pakistan with the published data mainly consisting of case reports. The gravity of the problem can be assessed from the emergence of repeated cases of poor-quality medicines reported for therapeutic failure [67] and from serious life-threatening conditions and fatalities which were eventually investigated with foreign technical assistance [19, 34]. All three prevalence studies involving analytical data were on antibiotics, among which two utilized testing according to compendial methods including (one international study sponsored by the innovator brand and other one as a publication from a PhD thesis). The third study was carried out at a local diagnostic laboratory and involved testing of various brands of ofloxacin from different regions within the country. The analytical studies were mainly research projects (PhD or M. Phil. theses) [70, 71, 74] showing divergent results, i.e. one study depicting no quality failures and two studies reporting a poor quality of the samples analyzed. Academia holds a huge potential for the conduct of such studies, but appropriate technical and financial support is required to develop quality evidence on the subject.

Public accessibility of drug testing data is advocated to promote transparency and to display the status of availability of quality medicines in the country [38]. Regarding statistics on quality evaluation and dissemination of safety alerts, the progress of PDCU is exemplary as there are no earlier instances of public sharing of such information in Pakistan [59]. However, these figures only represent limited statistics shared publicly by one province.

It is to be noted here that the major portion of the samples tested in the public sector laboratories was received from the tender supplies of public sector health facilities. Moreover, terms used in these reports to denote poor-quality medicines are inconsistent (substandard, spurious, unregistered etc.), thus a more scientific approach needs to be maintained in the reporting of official data in this respect. Authors suggest the regulators to use the internationally accepted terms of substandard and falsified medicines along with the separate term to describe the unregistered medicines (medicines without market authorization), and ambiguities in the legal terms provided in the Drug Act 1976 [49] also need to be resolved on the basis of current evidence and practices of WHO. Furthermore, these reports should be considered under the technical capacity of the operating DTLs. Even after the recent improvement in the system these laboratories vary in their technical capacity to perform complete compendial testing which only can assure that the product is of standard quality. The discussed reports are based upon limited quality evaluation including physical tests, assay, disintegration, and dissolution tests carried out at the DTLs.

Complete compendial testing including impurity tests is not yet covered under the current infrastructure. Of note, analysis of the national data of recalls by stringent regulatory systems (the UK and Canada) shows that contamination (referred to out-of-limit content for impurities and the presence of microbial contamination), stability, and packaging defects are the most frequently reported quality failures [74, 75]. In light of these figures from well-regulated and resourced regulatory facilities, it can be presumed that the countries omitting impurity testing from the routine quality tests may have a higher rate of quality failure than what is reported in the national data. This practice weakens the regulatory controls, as both industry and regulatory agencies neglect the conduct of impurity profiling placing the population at the risk of possible safety issues. The two major cases from Pakistan on medicine quality are also the result of such neglect [35, 66]. Resource limitation and skilled human resources are the major reasons given for this deliberate omission. It is important to note here that some of the monographs (e.g., assay and impurity testing method for ceftriaxone powder for injection described in the British Pharmacopoeia (2018) and the United States Pharmacopoeia (42nd Edition) offer assay and impurity testing using the same method. Ceftriaxone is a an important and extensively used essential medicine, thus the lack of reporting on impurity testing of this molecule is cannot be justified as the laboratories are already conducting the assay using the same method.

Various barriers and facilitators can be identified from the limited information which is available for Pakistan, e.g. the lack of technical capacities in the field of modern approaches to combat SF medical products. The key funding institutions like the Higher Education Commission (HEC) or the Central Research Fund (CRF) of Pakistan did not yet identify the issue of medicine quality being of priority for research funding. The Higher Education Commission funds scientific, technical, and policy research aimed to ensure that the country is able to meet standards of technology and practice required to progress to developed nations. HEC must ensure that the curriculum for professional programs (pharmacy and related fields) and the institutional facilities are optimum for training and capacity building in accordance with international standards. On the other hand, international agencies like the WHO and the USP “promoting the quality of medicines” program are actively supporting capacity building of regulators and industry personnel on the subject [39]. However, the lack of involvement and ownership of stakeholders, especially from academia, research and funding bodies, creates a major gap. Collaboration of these stakeholders is crucial to develop sound evidence that can inform policy in a productive manner. Field studies, the use of diverse technological approaches, and studies on development and evaluation of tools for detection and control of SF medicines are important areas that can be led by academicians and researchers.

Evidence based policies are of crucial need in making the correct choices in technology as well as developing the technical capacity to investigate issues like adulterants, pharmaceutical impurities, and performance failures. Researchers with policy, regulatory and analytical expertise work closely together to develop such evidences. International literature describes a rich body of data on the prevalence of poor-quality medicines, mainly from sub-Saharan Africa where the availability of basic field data had resulted in an increased sensitivity to the issue and has driven resources to device solutions with internal and external initiatives [76].

### Recommendations for Pakistan

A set of recommendations for Pakistan based on the gaps identified during the review process and the general country settings of Pakistan is provided below. Lessons from LMICs and important resources of cost-effective technological approaches are also incorporated.

### Capacity building

Academics, professionals, and researchers in the field of pharmaceutical regulation require training on modern analytical techniques for quality evaluation of pharmaceuticals and experts as well as skilled human resources need to be developed in order to establish a stringent regulatory system. Objective-oriented undergraduate training, specifically addressing the areas on quality evaluation, pharmacovigilance, international regulatory guidelines and pharmaceutical policy must be ensured.

### Participation in international forums

Membership and effective participation in international forums can help in capacity building of the national regulatory authorities and the drug testing laboratories. In 1995, the European Directorate for the Quality of Medicines (EDQM) set up a Network of Official Medicines Control Laboratories (OMCLs) which is partially funded by the European Commission and works through resource pooling by the competent national laboratories [77]. The annual agenda of the network is formulated in consultation with the national laboratories to support the regulatory authorities for the control of quality of marketed medicinal products both of human and veterinary origin, respectively [77]. Currently, four other networks are operating in different regions of the world including the External Quality Control Programs (EQCP) network, which includes the Pan American Health Organization (PAHO) and OMCLs from Latin American and Caribbean countries; so do the Networks of Official Medicines Control Laboratories (NOMCoL) in Africa, Middle East/North Africa (MENA), and Asia Pacific [78]. A similar initiative is proposed at the regional level addressing the needs of Pakistan and its neighboring countries.

### Improved infrastructure

Mobilization of public and private sector funding for fulfilling essential infrastructure needs and ensuring resource sharing, national and international collaboration and out-sourcing for the missing facilities are important steps that can be taken to develop a cost effective, efficient and sustainable system for regulatory and drug testing infrastructure. Collaboration with private sector and hi-tech research centers and universities can be a promising option in this regard. Lack of proper infrastructure for drug testing has also been discussed earlier as well for Pakistan in various reports including the investigations on the Fake Drug Crisis mentioned earlier in the article [19, 34]

### Emphasis on information technology-driven accessible technologies for quality control of pharmaceuticals developed for low- and middle countries

Pakistan is not yet part of any initiative regarding the use of evolving technologies and new approaches in fighting the menace of SF medicines and has been solely relying on the conventional approach of compendial testing. It has already been pointed out that due to the lack of technically skilled human resources and financial constraints, these compendial testing procedures have not been and are not carried out in a comprehensive manner. Regulators, technical staff, and policy makers need to be aware of the current scientific trends and the related evidence regarding use and choice of technologies. A variety of technologies and approaches has been published for detecting falsified and substandard medicines in various constraint setting [79–82] and a comparison of the technologies based on cost-efficiency, simplicity, and performance was also made [79, 80, 82]. Many of the tools suitable for use in LMICs are completely or at least partially based upon information technology as already reviewed by us [83]. Advancement in the surveillance of poor-quality medicines cannot be achieved with the isolated and redundant approach. Cost-effective and evidence-based approaches are of crucial importance in the design of surveillance programs aimed at detection and control of SF medicines at national level especially in LMICs.

### Rapid and cost-effective field-testing techniques

Field testing has been successfully adopted as part of the surveillance programs of LMICs. These testing procedures involve using low-cost handheld devices which require limited skills for operation and are excellent for testing carried out in remote regions and constrained settings. Apart from improving the detection of quality issues, the approach is also capable of filtering a major portion of the samples before being transferred to drug testing laboratories, thus reducing their overall sample load [82]. This demands a revamping of the infrastructure. Lessons must be learned from the countries carrying out a similar scheme of operation, e.g. China and sub-Saharan Africa. Africa has been the focus of many technological initiatives regarding fast and efficient detection, field testing, and reporting of poor quality medicines [84] including the German Minilab® project which has been successfully run in the continent since many years and which has produced significant impact in the reporting of substandard and falsified medicines.

### Use of intermediary test methods (simple and cost-effective analytical methods) for assay and impurity profiling of essential medicines identified for quality failures

Fast, simple, and cost-effective HPLC methods for about ten antimalarial drugs have been developed and validated for assay and impurity profiling [82, 85, 86]. These methods aim to provide simplicity, reliability, and efficiency to pharmaceutical testing processes at regulatory and quality control laboratories [82]. Using generic, intermediary HPLC methods, improved analytical facilities, and larger capacities to test more samples had consistently set a deterrent to the counterfeiters over a number of years [82].

### Risk based post marketing surveillance (PMS)

Strategic sampling strategies like risk-based post marketing surveillance need to be employed. A proposal of a risk-based surveillance program for Pakistan has been developed and presented lately [87]. Risk-based PMS has successfully been tested in sub-Saharan African countries [81]

### Transparency and public access to surveillance data

Understanding of the quality issues of medicines by the society can be enhanced by increasing public access to surveillance data on quality of medicines. Currently, the large body of data lies with either Industry or with the regulatory laboratories. Transparency and public access to information is also advocated for steps to ensure the availability of quality essential medicines needed for Universal Health Coverage [38]. These actions built the public confidence on the health care system and act as a deterrent to people involved in mal practices of falsified medicines.

### Implementing the WHO prequalification system

The WHO has devised a system to ensure safety of the supply chain that prevents infiltration of poor-quality products through tender or large volume supplies [32]. Manufacturers are warned of serious consequences if their product is found to be substandard. E.g., the prequalification program helped Kenya to reduce the quality failures of medicines from 35 to 40% to 3–5% as an impact to the strict surveillance procedures by the Mission for Essential Drugs and Supplies (MEDS). MEDS is a non-governmental organization which runs a WHO accredited quality control laboratory [38].

### Local bioequivalence study centers

Bioequivalence studies were introduced in 1984 in the USA as a requirement to prove generic equivalence. Intravenous medicaments and bio-waivered classes of medicines are technically exempted. Mandatory bioequivalence for product registration is yet to be effectively implemented in many parts of the world like India and Pakistan as the two countries have recently initiated regulations in this regard. Four out of thirty-four bioequivalence studies conducted on different antimicrobial agents showed that the generic medicines (comparator) were significantly inferior to the innovator brand, the rest proved otherwise [88]. The WHO recommends only using bioequivalent products for the fixed dose combinations used in the tuberculosis control programs. In 2009, the Bioequivalence Study Centre at the University of Veterinary and Animal Sciences came up as the first CRO to obtain approval from DRAP for carrying out respective studies. Among the seven bioequivalence study centers that received DRAP registration [89], none is functional due to reasons like awaiting renewal and adoption of new biostudy rules by DRAP. Non-availability of fully functional bioequivalence centers in the country pose hurdle in the adoption of mandatory bioequivalence of pharmaceutical products, a step that can indirectly improve the quality of products manufactured locally in the country as well as offer significant cost advantage by promoting generic medicines. DRAP should ensure functioning of such study centers so as to improve manufacturers’ compliance to the mandatory bioequivalence for the products that do not hold bio-waiver.

### Improving national GMP standards

A level close to the global GMP requirements set by WHO and an adoption of internationally accepted standard definitions regarding quality failures of medicines will be important steps for ensuring successful interventions. The need of improvement in GMP compliance has been highlighted in previous studies as well [57, 73].

### Emphasis on research related to medicine quality issues

Adequate funding and promotion of the research on quality issues with regional and international collaboration for capacity building and resource sharing in this regard should be carried out [38]. Promotion of research in academic institutions should be achieved through the development of focused research centers and declaration of SF medicinal products as one of the priority research areas in public sector research funding [76]. Industry-academia or private-public partnerships for post-marketing surveillance should be encouraged

### Developing and strengthening of national pharmacovigilance systems

Implementing and encouraging voluntary recalls of substandard medicines identified by mechanisms like PMS, ADR reporting and action on suspicious medicines, need to be established. Unlike developed health care system, in LMICs a huge barrier is seen in practicing of reporting of ADRs, volunteer recalls something which is considered a routine activity in stringent regulatory systems like FDA. Efforts must be made to develop a positive and healthy perception of society towards “reporting” and “recalls” of SF medicines to make the newly established pharmacovigilance system effective. Awareness of public and health care professionals on safe-guarding against poor quality medicines must be raised through seminars as well as use of print, electronic and social media. The approach will not only result in timely identification and reporting of SF medicines but will also public aware of the risk involved with purchase of medicines from unreliable supply chains.

### Effective communication with health care professionals

Adopting simple and efficient concepts for communicating safety alerts and establishing a pharmacovigilance system is crucial to a timely identification of poor-quality medicines within the supply chain. Some simple and practical approaches are used in the industrialized world. In Germany for example, the mechanism of “Rote-Hand-Brief” (“Red Hand Letter” or “Dear Doctor Letter”) is used as a safety alert tool. It is issued by the BfArM and the Paul-Ehrlich-Institute (PEI) to communicate any information of safety concerns regarding drugs, medicinal products, vaccines, and biologicals requiring immediate action [79]. The letters show a pictogram of a red hand symbolizing “stop”, thus highlighting the relevance of the underlying information. After any medicine related quality issue or adverse drug reaction has been observed, health care team members can report e.g. to the “Arzneimittelkommission der Deutschen Apotheker” (AMK) through different channels. The issuance of a “Rote-Hand-Brief” can be eventually triggered. Registered pharmacies and practitioners have access to the on-line software “Identa®” [90] for verifying physical properties of the medicaments which helps in the early identification and reporting of falsified medicines in the market. Regular and randomly selected package checking is performed on pharmacy stocks by a pharmacist as a mandatory recurring procedure to identify obviously visible quality defects.

### Strengthening reporting and recall systems

Setting a category of the respective recall ensures triggering of appropriate response from the respective target audience. “Health Canada” issues the risk communication under five categories applicable to medicine quality. These recalls are further divided into type I-III according to the severity and urgency of the recall [75]. Drug alerts issued by the MHRA are classed in four categories with class 4 not being intended for recall and only needs “caution in use” [74]. A format suited for engagement of local stakeholders can be initiated in Pakistan.

### National action plan

A comprehensive multi-component and multidisciplinary National Action Plan should be devised by involving various stakeholders to combat the issue of poor-quality medicines in Pakistan.

### Collaboration with other LMICs involved in fighting poor-quality medicines

Clinical failure and increasing resistance to malaria therapy served as a driving force to combat poor quality of medicines in Africa since the 1990s. A regional movement was started with antimalarials to which antituberculous and anti-retroviral drugs were added in a later phase. Rwanda achieved the lowest incidence of substandard and falsified drugs in Africa. It attributes its success to an improved country’s supply chain, access to high quality medicines in the public sector, and drug surveillance system. Coordinated inspections, verifications, and release procedures by the multidisciplinary teams are employed for importing medicines [55, 91]. A well networked pharmacovigilance system is established containing more than 2400 trained workers [55]. Nigeria has reduced the prevalence of counterfeit, substandard and falsified drugs from 41 to 80% to about 16% [92] by controlling medicines before crossing the outer borders [93]. They ensure the provision of pre-shipment information by the manufacturers/exporters and depute analysts in the source country like India to ensure testing of medicines prior to import. Höllein et al. have discussed the two decades journey of the Tanzanian Food and Drug Authority (TFDA) in bringing down the number of cases of poor-quality medicines, with its systematic interventions, scientific approach, planning, and international collaboration [82].

## Conclusion

This review has outlined the country situation for Pakistan and the options it has for embarking on the journey to fight against poor-quality medicines. The case reports and investigations collected for Pakistan are suggestive of the need to strengthening of the regulatory systems for premises and GMP inspections, strengthening analytical laboratories as well as the capacity building on the overall area of controlling substandard and falsified medicine in Pakistan. The figure of 40–50% of poor-quality drugs in Pakistan cannot be defended by the available literature. It is proposed that systematic objective data needs to be developed through well planned funded studies for collecting critical statistics regarding substandard and falsified medicinal products in Pakistan. The country is progressing towards an improved regulatory structure at a fast pace but a comprehensive and long-term vision with multidisciplinary, open, progressive and evidence-based approach is needed for successful transition towards a well-regulated system.

## Data Availability

The datasets and information used and analyzed during the current study are available from the corresponding author on request.

## Abbreviations

AMK: Arzneimittelkommission der Deutschen Apotheker
API: Active Pharmaceutical Ingredient
BfArM: Federal Institute for Drugs and Medical Devices (German Bundesinstitut für Arzneimittel und Medizinprodukte)
CRF: Central Research Fund
DCO: Drug Control Organization
DRAP: Drug Regulatory Authority of Pakistan
DSA: Drug Safety Alerts
DTL: Drug Testing Laboratory
FDA: United States Food and Drug Administration
FTDA: Tanzania Food and Drug Authority
GMP: Good Manufacturing Practices
HEC: Higher Education Commission
HPLC: High Performance Liquid Chromatography
ISO: International Organization for Standardization
LMIC: Low- and Middle- Income Countries
MEDS: Mission for Essential Drugs and Supplies
MHRA: Medicines and Healthcare products Regulatory Agency of the UK
OMCLs: Official Medicines Control Laboratories
PDCU: Provincial Quality Control Unit of Punjab
PDTRC: Punjab Drug Testing Laboratory and Research Centre
PEI: Paul-Ehrlich-Institut
PIDM: Programme for International Drug Monitoring
PMS: Post Marketing Surveillance
SF: Substandard and falsified
TLC: Thin Layer Chromatography
US-$: United States Dollar
USP: United States Pharmacopoeia
WHO: World Health Organization
